# Neurotrophic Role of the Next-Generation Probiotic Strain *L. lactis* MG1363-pMG36e-GLP-1 on Parkinson’s Disease via Inhibiting Ferroptosis

**DOI:** 10.3390/nu14224886

**Published:** 2022-11-18

**Authors:** Mengyun Yue, Jing Wei, Wenjie Chen, Daojun Hong, Tingtao Chen, Xin Fang

**Affiliations:** 1 Department of Neurology, The First Affiliated Hospital of Nanchang University, Nanchang 330006, China; 2 National Engineering Research Center for Bioengineering Drugs and Technologies, Institute of Translational Medicine, Nanchang University, Nanchang 330031, China; 3 Queen Mary School, Jiangxi Medical College, Nanchang University, Nanchang 330031, China; 4 Nanchang Joint Programme in Biomedical Sciences, School of Biological and Chemical Sciences, Queen Mary University of London, London E1 4NS, UK

**Keywords:** Parkinson’s disease (PD), *L. lactis* MG1363-pMG36e-GLP-1, glucagon-like peptide-1 (GLP-1), ferroptosis, oxidative stress, dysbiosis

## Abstract

Parkinson’s disease (PD) is a neurodegenerative disease (NDD) with high and ongoing morbidity, bringing heavy burdens to PD patients seriously. Finding neurotrophic drugs still remains vital due to the limited drug spectrum available currently. Substantial evidence suggests that glucagon-like peptide 1 (GLP-1) exerts neuroprotection on PD, yet the short-lived biological activity markedly hindered its application. Herein, we investigated the neurotrophic role of the next-generation probiotic strain *L. lactis* MG1363-pMG36e-GLP-1 in 1-methyl-4-phenyl-1,2,3,6-tetrahydropyridine (MPTP)-induced PD mice and elucidated the mechanisms. Our data suggested that *L. lactis* MG1363-pMG36e-GLP-1 markedly enhanced motor deficits induced by MPTP via rescuing dopaminergic (DAergic) neurodegeneration in substantia nigra (SN). We found that *L. lactis* MG1363-pMG36e-GLP-1 exerts neurotrophic effects via activating the Keap1/Nrf2/GPX4 signalling pathway to down-regulate ACSL4 and up-regulate FSP1 to suppress ferroptosis. Additionally, the decreased oxidative stress levels via suppressing generations of ROS and MDA supported our findings. Lastly, we identified that the *L. lactis* MG1363-pMG36e-GLP-1 administration reversed dysbiosis in PD mice by increasing *Akkermansia*, *Oscillospira*, and *Sutterella* at the genus level. These results indicated that the neurotrophic effects of the next-generation probiotics *L. lactis* MG1363-pMG36e-GLP-1 against MPTP-induced Parkinsonism are mediated by modulating oxidative stress, inhibiting ferroptosis, and redressing dysbiosis.

## 1. Introduction

Parkinson’s disease (PD) is a kind of neurodegenerative disease (NDD) affecting over 6 million people worldwide, which is going to rise to 9.3 million by 2030 [1,2]. Clinically, PD is manifested as progressive locomotive disorder and cognitive impairment, which is thought to be associated with Lewy bodies (LBs) and dopaminergic (DAergic) neuronal death in the substantia nigra (SN) [3,4]. The high and ongoing prevalence of the disease brings heavy burdens to PD patients and society seriously. Current first-line PD treatment includes monoamine oxidase (MAO) inhibitors, levodopa (L-DOPA), anticholinergics, amantadine, and dopamine (DA) receptor agonists, all of which may improve symptoms but cannot prevent or reverse disease progression [5]. Moreover, adverse incidents such as nausea, somnolence, and orthostatic hypotension restrained their clinical use [6].

Ferroptosis refers to caspase-independent cell death characterised by intra-cellular iron sedimentation, which is extensively involved in pathological cell death in many diseases, especially NDDs [7,8]. It had confirmed that ferroptosis is correlated with iron deposition in the SN, causing glutathione (GSH) consumption, reactive oxygen species (ROS) increment, and DA oxidation, resulting in pathogenic changes in the PD mice [9]. Autopsies on PD patients also found the iron accumulation identified in DAergic neurons is highly involved in neuronal death-induced disease progression [10].

Glucagon-like peptide 1 (GLP-1) is a bioactive peptide produced by ileal enteroendocrine L cells, originally approved for treating type II diabetes and obesity [11,12]. In the recent decade, studies have reported neurotrophic effects of GLP-1 in the PD mice models via decreasing the DAergic neuronal death [13]. Additionally, GLP-1 analogues launched clinical trials in PD patients, which retrieved promising results [14,15]. However, the short half-life of GLP-1 mediated by transient enzymolysis from dipeptidyl peptidase IV (DPP-IV, CD26) restricted its clinical use. Consequently, replenishing continuously generated GLP-1 to outweigh the proteolysis of DPP-IV is considered a better strategy. In our former research, we demonstrated the pharmaceutical potential of GLP-1 in treating multiple diseases including diabetes [16], obesity [17], Alzheimer’s disease (AD) [18,19], and PD [19,20]. The previously constructed next-generation probiotics *Lactococcus lactis* (*L. lactis*) MG1363-pMG36e-GLP-1 was confirmed to steadily secrete GLP-1 as well as exerting neurotrophic effect on MPTP-induced PD model mice by derepressing tyrosine hydroxylase (TH) and reducing α-syn aggregation to improve motor functions [20]. However, the possible influence of GLP-1 on ferroptosis in PD sufferers is yet unknown.

Herein, we continued to study the neurotrophic role of next-generation probiotic strain *L. lactis* MG1363-pMG36e-GLP-1 on MPTP-induced PD mice model by intragastric administration and determined the neuroprotection and neurotrophy mediated by suppression of DAergic neuronal ferroptosis. The regulatory efficacy of the next-generation probiotic strain is quantified by ultrastructural pathology and histopathology. The changes in ferroptotic pathway-related proteins are detected by Western blotting. The alteration of gut microbiome is analysed by high-throughput sequencing.

## 2. Materials and Methods

### 2.1. Construction of the Next-Generation Probiotics

The next-generation probiotic strain used in this study was from lab stock, con-structed by Chen et al. [18]. Briefly, the GLP-1 gene sequence (1-37, GenBank: XM_023217335.1) was inserted into pMG36e to construct engineered plasmid pMG36e-GLP-1. After that, the transformed probiotic strain *L. lactis* MG1363-pMG36e-GLP-1 was constructed via electroporation of engineered plasmid [17,20].

### 2.2. Animals and Experimental Design

Fifty male C57BL/6 mice provided by Hunan SJA Laboratory Animal Co., Ltd. (Changsha, China) resided in an animal house (temperature 26 ± 1 °C, humidity 50 ± 10%), in which the light was on for 12 h and off for 12 h. Mice were acclimatised for 1 week and allowed water and animal food with no limitations. Then, all mice were stochastically divided into 5 groups using random number tables available online (https://www.random-online.com/, accessed on 26 December 2021), including: (1) C group, a control group treated with normal saline for 7 consecutive days (*n* = 10); (2) M group, a model group with intraperitoneal injection of 20 mg/kg/day MPTP (Sigma-Aldrich, Taufkirchen, Germany, M0896) for 7 consecutive days (*n* = 10); (3) L group, treated with MPTP and 0.4 mg/kg/day liraglutide for 7 consecutive days (*n* = 10); (4) R group, treated with MPTP and 10^9^ colony-forming unit (CFU) *L. lactis* MG1363 for 7 consecutive days via gavage (*n* = 10); (5) RG group, treated with MPTP and 10^9^ CFU *L. lactis* MG1363-pMG36e-GLP-1 for 7 consecutive days via gavage (*n* = 10). All animals survived treatment and all animal experiments were administered from 9:00 to 12:00 in the morning to reduce systematic errors.

### 2.3. Sample Collection

**Figure 1:**
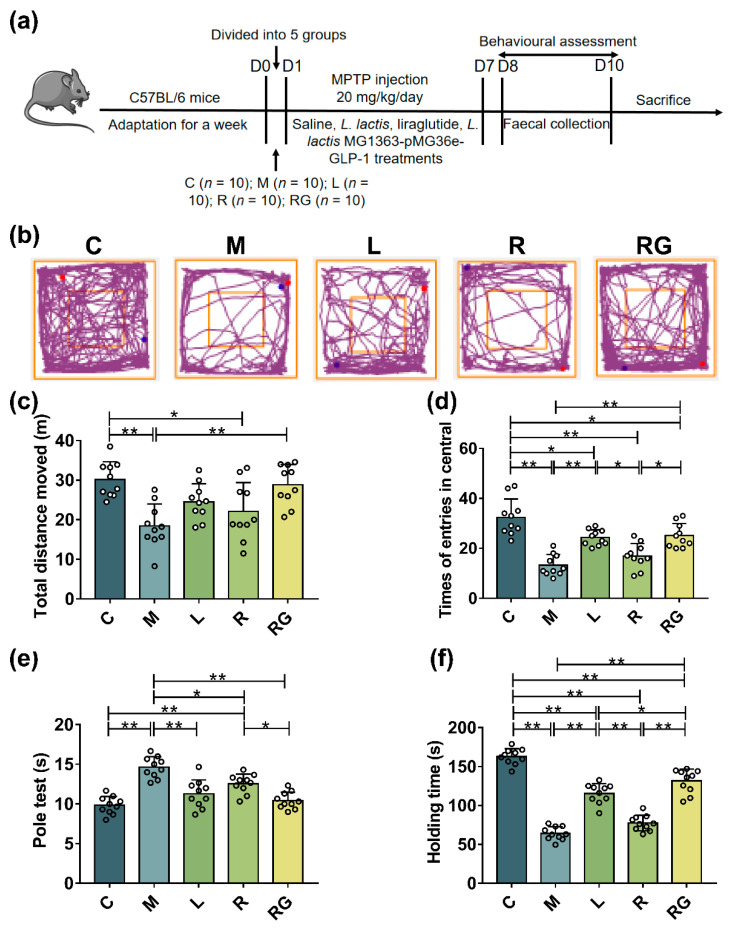
*L. lactis* MG1363-pMG36e-GLP-1 enhanced motor deficit in PD model mice. (**a**) Treatment timetable to explain experimental design of the animal experiment. (**b**) Representative traces of mice during open field test; blue point (●): beginning point; red point (●): end point. (**c**) The overall distance moved during open field test (*n* = 10). (**d**) The times of entries in central in the open field test (*n* = 10). (**e**) The pole test (*n* = 10). (**f**) The hanging wire test (*n* = 10). * *p* < 0.05 and ** *p* < 0.01.

Behavioural tests and faecal sample collecting were performed on days 8–10. At the end of treatment, the mice were sacrificed after anaesthesia with isoflurane gas [21]. Colon and brain tissues were collected immediately and then fixed for further histological staining. Plasma samples were obtained from centrifugation of blood at 3500 rpm for 20 min. Except for fixed samples, all other specimens were stashed at −80 °C for future experiments (Figure 1a).

### 2.4. Behavioural Experiments

The open field test and the pole test were performed as described in a former study [20]. The hanging wire test was performed as described by Li et al. [22]. The mice were trained to hang their bodies onto a steel wire (Φ = 2 mm) with their forelimbs only. The wire was dangled 30 cm above a soft cushion. The average holding time taken 3 times was recorded with 15 min intervals between trials to rest. Mice not able to hold the wire for more than 18 s after training would be excluded.

### 2.5. Ultrastructural Pathology

The ultrastructural changes of mitochondria were evaluated by transmission electron microscope (TEM). To prepare specimens for ultrastructural microscopy, the fresh SN tissue blocks were cut into granules smaller than 1 mm^3^. Then, the tissue was then immediately submerged in electron microscopy fixative (Servicebio, Wuhan, China, G1102) at 4 °C for later use. Next, the resin blocks were cut into 60–80 nm thick serial sections on an ultramicrotome before staining with uranyl acetate [UO_2_(CH_3_COO)_2_] and lead nitrate [Pb(NO_3_)_2_]. The ultrastructure was recorded using TEM.

### 2.6. Histopathology

Fixed tissues were embedded in paraffin after dehydration. The iron distribution in SN was mapped by Perl’s staining. After deparaffinisation, sections were rinsed with phosphate buffer saline (PBS), and the designated sample was incubated with 3% H_2_O_2_ for 20 min at room temperature (RT). Next, the sections were submerged into fresh Perls’ dye solution following diaminobenzene (DAB) staining and mounting. For H and E staining, the fixed tissues were dehydrated, embedded, and cut into 2 μm thick serial sections. After dewaxing, staining, dehydration, and clearing, the H and E staining tissues were sealed with a mounting medium.

Immunofluorescence and immunohistochemistry were performed as well. Briefly, the paraffin-embedded brain tissues were cut into 5 μm thick sections with microtome, deparaffinised, rehydrated, and incubated. For immunofluorescence and immunohistochemistry, the specimen was incubated with primary antibodies overnight at 4 °C (Supplementary Material Table S1). The secondary antibody was then incubated. All histopathological Figures were obtained under a light microscope.

### 2.7. Dihydroethidium (DHE) Staining

DHE staining was used to assess intracellular ROS levels. Concisely, the frozen sections were incubated with DHE (Servicebio, Wuhan, China, GDP1018) at RT for 30 min lucifugously, then rinsed three times with PBS (pH = 7.4) on a decolorizing shaker for 5 min each time. Next, 6-diaminidine-2-phenylindole (DAPI) was applied to the slightly dried slides and incubated for 10 min at RT lucifugously. Lastly, fluorescence intensity was recorded with a fluorescence microscope and quantified with ImageJ version 2022.

### 2.8. Western Blotting

Brain and colon tissues were homogenised before adding the prepared RIPA lysate containing protease inhibitor cocktail (Servicebio, Wuhan, China, HY-K0010), protein samples were then obtained by centrifugation at 9000 rpm for 20 min. Protein samples were separated by 8–15% sodium dodecyl sulfate-polyacrylamide gel electrophoresis (SDS-PAGE). The separated protein was then transferred onto polyvinylidene difluoride (PVDF) membranes (Millipore, Boston, MA, USA, IPVH00010). After blocking at RT for 90 min, the membrane was then incubated with primary antibodies (Supplementary Material Table S1). The membranes were incubated with a secondary antibody for 1 h at RT. Lastly, the activity of those specific proteins was detected by adding a proper amount of chemiluminescence solution (Thermo Fisher, Waltham, MA, USA, 32209) following exposed by a gel imaging system and quantified by ImageJ for later analysis.

### 2.9. Measurement of Oxidation-Associated Factors and GLP-1

The brain tissues and plasma samples were used to detect oxidation-associated factors levels, and total malondialdehyde (MDA), glutathione peroxidase (GSH-Px), and superoxide dismutase (SOD) were assessed using the assay kit provided by Nanjing Jiancheng Bioengineering Institute (Nanjing, China), following protocols offered by the manufacturer. The brain tissues were used to detect the GLP-1 levels via GLP-1 ELISA kit (Immuno-Biological Laboratories Co., Ltd., Tokyo, Japan, 27788), following protocols offered by the manufacturer.

### 2.10. 16S rRNA High-Throughput Sequencing

The bacterial genomic DNA was extracted from the DNA extraction kit (Tiangen, Beijing, China, DP712). The target fragments of the 16S rRNA V4 region were amplified using a universal primer for bacteria, and the amplification products were sequenced with the Illumina platform (San Diego, CA, USA). Then, the ASV/OUT signature sequences were obtained via the DADA2 method. According to ASV/OUT, the data of species composition, α-diversity, β-diversity, and species difference analysis were performed.

### 2.11. Statistical Analysis

Prism version 7.0 (GraphPad, San Diego, CA, USA) was used for data analysis. Numerical data are presented as means ± standard deviation (SD). Statistical analysis was conducted by one-way analysis of variance (ANOVA) followed by Tukey’s multiple comparison tests. Statistical significance was set at * *p* < 0.05, ** *p* < 0.01, and *** *p* < 0.001.

## 3. Results

### 3.1. L. lactis MG1363-pMG36e-GLP-1 Enhanced Motor Deficit in PD Model Mice

To assess the motor functions of the MPTP-induced PD mice model, relative behavioural tests were performed. During the open field test, the trace of mice explored is shown in Figure 1b. During the assay, mice in the M group exhibited less total movement distance compared with the C group, indicating that exploratory ability difference in M group is lower than C group (C vs. M = 30.19 m vs. 18.43 m, Figure 1c, *p* < 0.01), the times of entries in the central area was also reduced (C vs. M = 32.40 vs. 13.30, Figure 1d, *p* < 0.01). In addition, these parameters were improved moderately in PD mice treated with next-generation probiotic strain, wild-type probiotic strain, and liraglutide. Indeed, *L. lactis* MG1363-pMG36e-GLP-1 treatment showed a better outcome than that of *L. lactis* MG1363 strain and liraglutide. The pole test indicated that *L. lactis* MG1363-pMG36e-GLP-1 showed the same tendency. The ingestion of MPTP induced significant motor degeneration in M group than C group (C vs. M = 9.83 s vs. 14.63 s, Figure 1e, *p* < 0.01), which was substantially ameliorated by next-generation probiotic strain (10.43 s, *p* < 0.01). In the hanging wire test, MPTP markedly reduced holding time in M group than C group (C vs. M = 162.90 s vs. 64.23 s, Figure 1f, *p* < 0.01). Notably, *L. lactis* MG1363-pMG36e-GLP-1 treated mice in the RG group (131.60 s) performed better than mice in L group (L vs. RG = 115.70 s vs. 131.60 s, *p* < 0.05) and R group (R vs. RG = 77.63 vs. 131.60 s, *p* < 0.01). Collectively, these results suggested that next-generation probiotic strain could restore dyskinesia caused by MPTP.

### 3.2. L. lactis MG1363-pMG36e-GLP-1 Suppressed DAergic Neurons Death and Synucleinopathy in PD Model Mice

**Figure 2:**
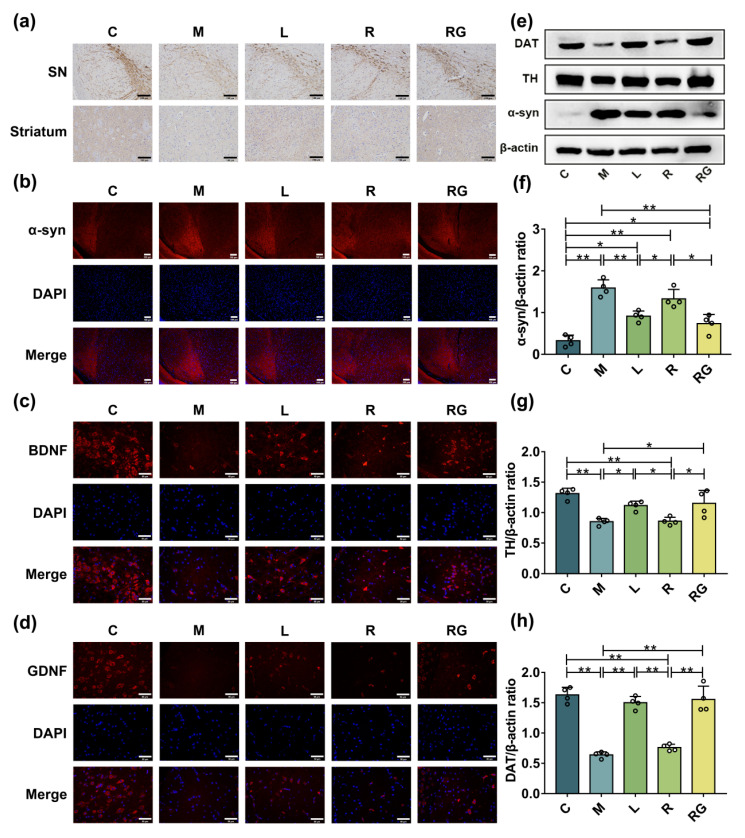
*L. lactis* MG1363-pMG36e-GLP-1 suppressed DAergic neurons death and synucleinopathy in PD model mice. (**a**) Photomicrographs of TH^+^ neurons in the SN and ST by IHC staining (scale bar = 100 μm). (**b**) Photomicrographs of α-syn in SN by IF staining (scale bar = 100 μm). (**c**,**d**) Photomicrographs of BDNF and GDNF in the SN of PD mice by IF staining (scale bar = 50 μm). (**e**) Western blotting of α-syn, TH, DAT, and β-actin in SN. (**f**–**h**) Relative quantification of α-syn, TH, and DAT protein levels adjusted to internal reference β-actin (*n* = 4). * *p* < 0.05 and ** *p* < 0.01.

To further study the neurotrophic role of *L. lactis* MG1363-pMG36e-GLP-1 on nigral and striatal DAergic neurons, immunohistochemistry, immunofluorescence, and Western blotting were used to access the survival of DA-positive (DA^+^) nerve cells. In our study, compared to the C group, the TH-positive (TH^+^) nerve cells in SN and striatum of the M group were decreased, while *L. lactis* MG1363-pMG36e-GLP-1 treatment greatly reduced neuronal loss (Figure 2a). Consistently, Western blotting of the SN further certified that the activity of TH in M group was decreased than C group (Figure 2e,g, *p* < 0.01) and this reduction could be reversed by *L. lactis* MG1363-pMG36e-GLP-1 administration (*p* < 0.05). Dopamine transporter (DAT), a plasma membrane protein, is one of the major DA modulators by transporting the released DA in the synaptic cleft back into the pre-synaptic membrane [23]. Western blotting of nigral tissue found that the activity of DAT in M group was reduced than C group (Figure 2e,h, *p* < 0.01) and this reduction could be reversed by *L. lactis* MG1363-pMG36e-GLP-1 treatment (*p* < 0.01). Another hallmark of PD is α-syn, which is increased in the M group determined by immunofluorescence staining (Figure 2b) and Western blotting (Figure 2e,f, *p* < 0.01) in the SN. The *L. lactis* MG1363-pMG36e-GLP-1 treatment reduced the α-syn aggregation (*p* < 0.01). Moreover, BDNF and GDNF could reduce DAergic neuronal death by promoting cell survival [24]. As shown in our study, *L. lactis* MG1363-pMG36e-GLP-1 restored the activity of BDNF (Figure 2c) and GDNF (Figure 2d) in PD mice models. In summary, our findings indicated that *L. lactis* MG1363-pMG36e-GLP-1 could improve histopathological changes of PD mice.

### 3.3. L. lactis MG1363-pMG36e-GLP-1 Increased GLP-1 and Potentially Improved the Integrity of the Blood-Brain Barrier (BBB) in PD Model Mice

**Figure 3:**
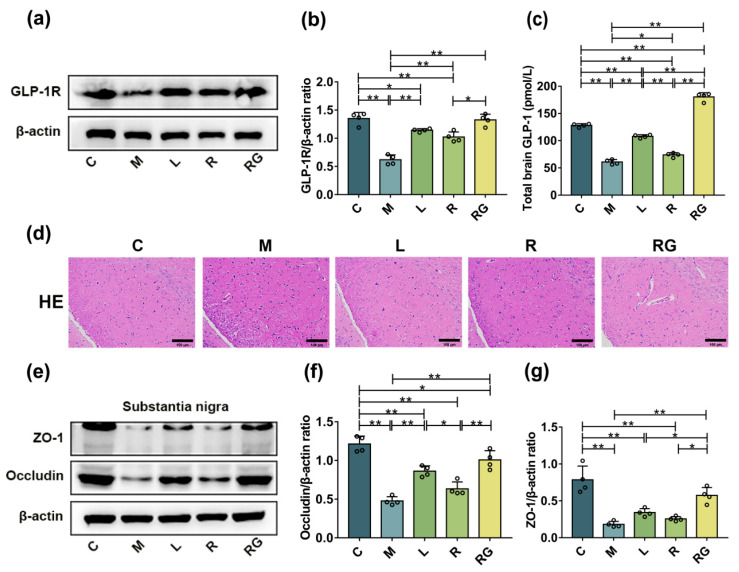
*L. lactis* MG1363-pMG36e-GLP-1 increased GLP-1 and potentially improved the integrity of the BBB in PD model mice. (**a**) Western blotting of GLP-1R and β-actin in the SN. (**b**) Relative quantification of GLP-1R protein levels adjusted to internal reference β-actin (*n* = 4). (**c**) GLP-1 concentrations in the SN as measured by ELISA (*n* = 4). (**d**) Images of H and E staining in the SN (scale bar = 100 μm). (**e**) Western blotting of ZO-1, occludin, and β-actin in the SN. (**f**,**g**) Relative quantification of ZO-1 and occludin protein levels adjusted to internal reference β-actin (*n* = 4). * *p* < 0.05 and ** *p* < 0.01.

We investigated the distribution of GLP-1 and its receptor in SN of PD mice by ELISA and Western blotting. The M group expressed lower levels of GLP-1R than the C group (*p* < 0.01), however, treatment with liraglutide (*p* < 0.01), MG1363 (*p* < 0.01), and *L. lactis* MG1363-pMG36e-GLP-1 (*p* < 0.01) inhibited the down-regulation of GLP-1R induced by MPTP (Figure 3a,b). Moreover, we found that the effect of the next-generation probiotic strain was stronger than wild-type *L. lactis* MG1363 (*p* < 0.05). As shown in Figure 3c, the GLP-1 concentration in the SN were measured, *L. lactis* MG1363-pMG36e-GLP-1 can prominently increase expression levels of GLP-1 in MPTP-induced PD model mice. Furthermore, it had stronger activity than liraglutide (*p* < 0.01). Since it is suggested that the integrity loss of BBB is crucial in pathogenesis of PD [25], we performed H and E staining to evaluate pathological changes and assessed the expression of key proteins in the barrier-associated proteins in the SN of PD mice using Western blotting. As shown in Figure 3d, *L. lactis* MG1363-pMG36e-GLP-1 treatment attenuated PD-related epithelial damage in the SN. Western blotting results also showed that the two major tight junction proteins were markedly decreased in the M group, including occludin and ZO-1 (*p* < 0.01) by comparison to the C group whereas *L. lactis* MG1363-pMG36e-GLP-1 treatment could restore such reductions (*p* < 0.01) (Figure 3e–g).

### 3.4. L. lactis MG1363-pMG36e-GLP-1 Inhibited Ferroptosis via Potentiating the Keap1-Nrf2-GPX4 Pathway in PD Model Mice

**Figure 4:**
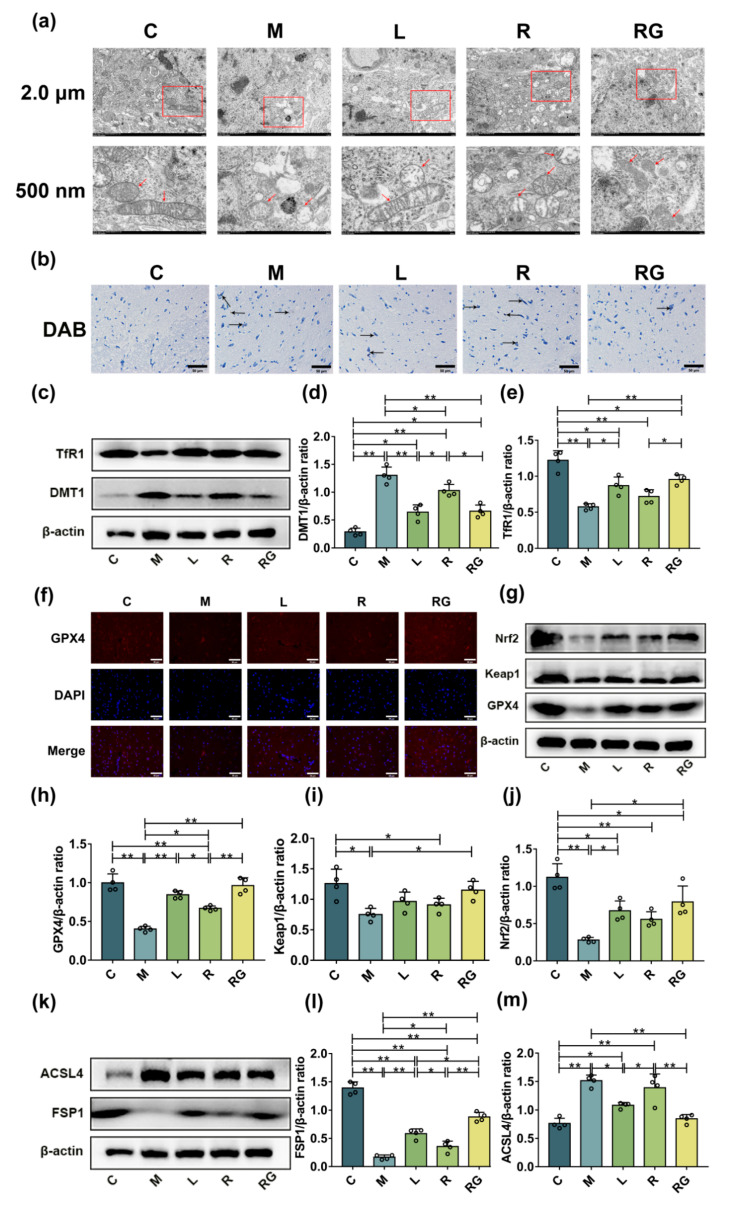
*L. lactis* MG1363-pMG36e-GLP-1 inhibited ferroptosis via potentiating the Keap1-Nrf2-GPX4 pathway in PD model mice. (**a**) Ultrastructure of mitochondria in SN of PD model mice as measured by TEM; the red box and arrow indicated mitochondria (scale bar = 2.0 μm or 500 nm). (**b**) Perls’ staining image of SN in PD model mice; the black arrow indicated iron deposition (scale bar = 50 μm). (**c**) Western blotting of TfR1, DMT1, and β-actin in SN. (**d**,**e**) Relative quantification of TfR1 and DMT1 protein levels adjusted to internal reference β-actin (*n* = 4). (**f**) Representative photomicrographs of GPX4 in the SN of PD mice as measured by IF staining (scale bar = 50 μm). (**g**) Representatives Western blotting of GPX4, Keap1, Nrf2, and β-actin in the SN. (**h**–**j**) Quantification of GPX4, Keap1, and Nrf2 protein levels adjusted to β-actin (*n* = 4). (**k**) Representative Western blotting of FSP1, ACSL4, and β-actin in the SN. (**l**,**m**) Relative quantification of FSP1 and ACSL4 protein levels adjusted to β-actin (*n* = 4). * *p* < 0.05 and ** *p* < 0.01.

We accessed whether next-generation probiotic strain can reduce mitochondrial damage-based cell death, namely ferroptosis. Compared with C group, ultrastructural pathology of PD mice showed that the mitochondria were diminished numerically and dwindled morphologically, which is reckoned as the major characteristic of ferroptosis, while *L. lactis* MG1363-pMG36e-GLP-1 treatment restored number and morphology of mitochondria (Figure 4a).

It was recently reported that GLP-1 showed great inhibitory effect on neuronal iron overload and ferroptosis [26]. Hence, we investigated the role of *L. lactis* MG1363-pMG36e-GLP-1 treatment on iron deposition and the activity of iron transporters in PD mice. As shown in Figure 4b, in contrast to normal mice, the quantity of iron-positive cells in SN was higher in PD mice by Perls’ staining. On the contrary, next-generation probiotic strain treatment suppressed the increase of iron-positive cells in SN. This suggested that the protection of *L. lactis* MG1363-pMG36e-GLP-1 can be associated with reducing iron deposition in PD model mice. Consistently, we investigated the underlying mechanism of iron absorption and release by determining the activities of iron transporter DMT1 and TfR1 using Western blotting. As shown in Figure 4c–e, the M group overexpressed DMT1 more than the C group (*p* < 0.01), whereas liraglutide (*p* < 0.01), *L. lactis* MG1363 (*p* < 0.05), and *L. lactis* MG1363-pMG36e-GLP-1 (*p* < 0.01) treatment inhibited the up-regulation of DMT1 due to absorption of MPTP. In addition, the activity of TfR1 was decreased after MPTP injection (*p* < 0.01), and *L. lactis* MG1363-pMG36e-GLP-1 (*p* < 0.01) notably renovated the activity of TfR1 in PD models. Thus, the impact of *L. lactis* MG1363-pMG36e-GLP-1 on suppression to iron deposition in SN of PD model mice is mediated by regulating iron transporter protein DMT1.

Then, we further investigated the ferroptotic pathway through which the *L. lactis* MG1363-pMG36e-GLP-1 exerted its ferroptosis-inhibitory effect. GPX4 is a necessary selenoprotein that suppresses lipid hydroperoxides on the membranes, suggesting its dominating enzymatic role in the ferroptotic pathway [27]. As shown in our study, GPX4 activity was markedly down-expressed in the SN of the M group (*p* < 0.01). However, the L (*p* < 0.01), R (*p* < 0.05), and RG (*p* < 0.01) groups restored MPTP-induced down-regulation of GPX4 in comparison with the M group (Figure 4g,h). The immunofluorescence of GPX4 in the SN was in accordance with Western blotting results (Figure 4f). In addition, we found that the Nrf2-Keap1 pathway was activated with the administration of *L. lactis* MG1363-pMG36e-GLP-1. The Nrf2-Keap1 pathway provides the major defence against oxidative stress by upregulating antioxidant enzymes [28]. As shown in Figure 4g,i,j, compared with C group, we found that the activity of Nrf2 (*p* < 0.01) and Keap1 (*p* < 0.05) were reduced in SN of PD model mice, while *L. lactis* MG1363-pMG36e-GLP-1 restored the levels of Nrf2 and Keap1 in PD mice (*p* < 0.05). Our findings indicated the neurotrophic effect of *L. lactis* MG1363-pMG36e-GLP-1 can be associated with regulating ferroptosis via activating the Keap1-Nrf2-GPX4 pathway.

Ferroptosis, a non-apoptotic iron-dependent cell death, had been found to be implicated in the pathophysiology of PD [29]. To further investigate whether *L. lactis* MG1363-pMG36e-GLP-1 can inhibit ferroptosis, we detected the ferroptosis-associated protein expression including ACSL4 and FSP1. As shown in Figure 4k–m, the data revealed that the expression of ACSL4 was upregulated, whereas there is a significant reduction of FSP1 in PD mice (*p* < 0.01). Nevertheless, *L. lactis* MG1363-pMG36e-GLP-1 recovered MPTP-induced up-regulation of ACSL4 and decrement of FSP1 (*p* < 0.01).

### 3.5. L. lactis MG1363-pMG36e-GLP-1 Reduced Systematic and SN Oxidative Stress in PD Model Mice

**Figure 5:**
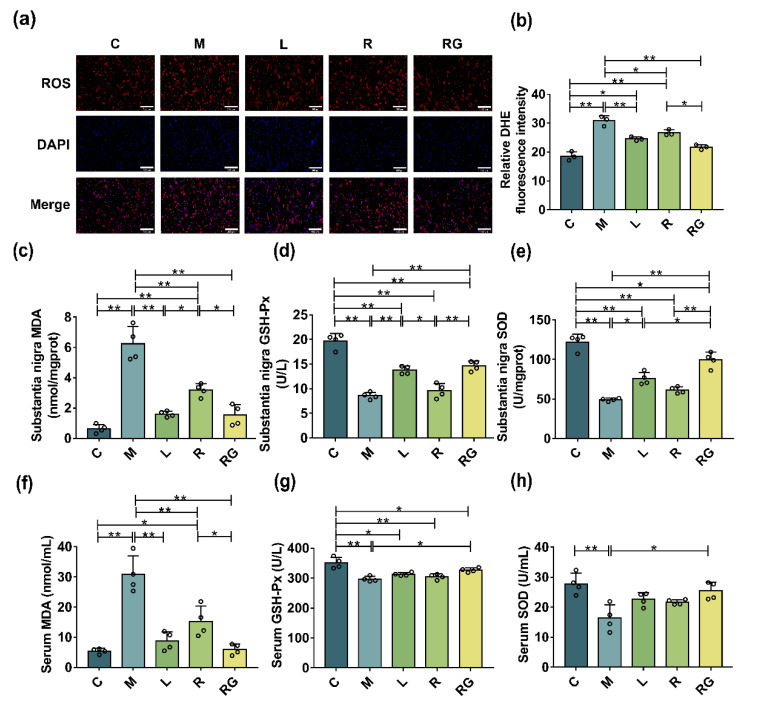
*L. lactis* MG1363-pMG36e-GLP-1 reduced systematic and SN oxidative stress in PD model mice. (**a**) Levels of ROS in SN of PD model mice as measured by DHE staining (scale bar = 100 μm). (**b**) The relative quantitation of florescent intensity of DHE staining (*n* = 3). (**c**–**h**) The levels of oxidative stress factors MDA, GSH-Px, and SOD in SN and serum of PD mice (*n* = 4). * *p* < 0.05 and ** *p* < 0.01.

Since excess iron is associated with ferroptosis and could aggravate ROS and lipid peroxidation [30], we further assessed the level of ROS in the SN of PD models (Figure 5a,b). The excessive ROS levels were eliminated by *L. lactis* MG1363-pMG36e-GLP-1 to a certain extent, which is detected by DHE staining.

We then assessed the positive effects of *L. lactis* MG1363-pMG36e-GLP-1 on oxidative stress in serum and SN of PD mice. In our study, we detected oxidative-related factor levels including MDA, GSH-Px, and SOD factors with corresponding assay kits. As shown in Figure 5c–h, we found that the total MDA content was up-regulated in the PD mice (*p* < 0.01), and *L. lactis* MG1363-pMG36e-GLP-1 reduced MDA levels both in the serum and SN (*p* < 0.01). Moreover, SOD and GSH-Px levels were lower in M group by contrast to the C group (*p* < 0.01). This downward trend was reversed after treatment with *L. lactis* MG1363-pMG36e-GLP-1, suggesting that *L. lactis* MG1363-pMG36e-GLP-1 restored the antioxidant abilities in the MPTP-induced PD mice.

### 3.6. L. lactis MG1363-pMG36e-GLP-1 Enhanced Intestinal Barrier and Reversed Dysbacteriosis in PD Model-Mice

**Figure 6:**
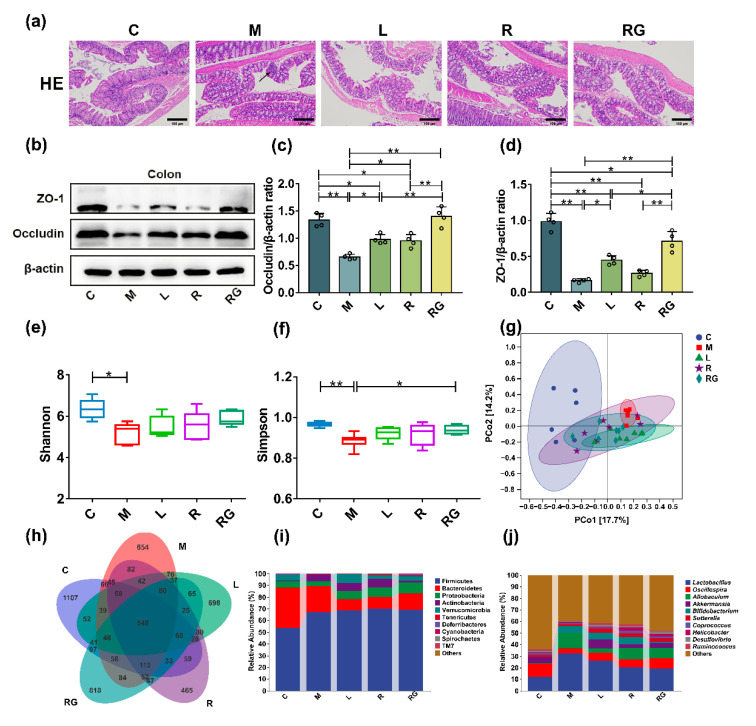
*L. lactis* MG1363-pMG36e-GLP-1 improved intestinal barrier and reversed dysbacteriosis in PD model mice. (**a**) Representative images of H and E staining in colon; →: inflammatory infiltration (scale bar = 100 μm). (**b**) Western blotting of ZO-1, occludin, and β-actin in colon. (**c**,**d**) Relative quantification of ZO-1 and occludin protein levels adjusted internal reference to β-actin (*n* = 4). (**e**) Shannon index. (**f**) Simpson index. (**g**) PCoA analysis. (**h**) Venn chart. (**i**) Abundance analysis at phylum level. (**j**) Abundance analysis at genus level. C group (*n* = 6), M, L, R, and RG group (*n* = 7). * *p* < 0.05 and ** *p* < 0.01.

In previous studies, it was confirmed that the integrity loss of intestinal barrier is a non-negligible factor in pathogenesis of PD [25]. Hence, we performed H and E staining to access the pathological changes and Western blotting of the intestinal tissue to determine the activity of key proteins of barrier-associated proteins in MPTP-induced PD model mice. *L. lactis* MG1363-pMG36e-GLP-1 treatment significantly attenuated PD-related inflammatory infiltration and epithelial damage in the colon (Figure 6a). Furthermore, the Western blotting also identified decreased activity of the two major tight junction proteins in M group, including ZO-1 (*p* < 0.01) and occludin (*p* < 0.01) than the C group whereas *L. lactis* MG1363-pMG36e-GLP-1 treatment markedly restored such reductions (*p* < 0.01) (Figure 6b–d).

We analysed the intestinal microbiota changes in experimental mice using 16S rRNA high-throughput sequencing. Shannon index (*p* < 0.05) and Simpson index (*p* < 0.01) were used to estimate the α-diversity of microbial communities, the M group markedly decreased the diversity of intestinal microbiota compared with the C group. Simpson index indicated that *L. lactis* MG1363-pMG36e-GLP-1 (*p* < 0.05) treatment in the RG group could ameliorate the neurotoxic effects of MPTP (Figure 6e,f). Furthermore, the principal coordinates analysis (PCoA) suggested that bacterial diversity altered in the M group, while such dysbacteriosis was reversed by *L. lactis* MG1363-pMG36e-GLP-1 treatment (Figure 6g). Next, the Venn results showed that 548 common OTUs were discovered in all of these five groups, and the unique OTU numbers discovered in groups C, M, L, R, and RG were 1107, 654, 698, 465, and 818, respectively (Figure 6h).

At the phylum level, the abundance of Firmicutes, Actinobacteria, and Tenericutes was increased, whereas the abundance of Bacteroidetes, Proteobacteria, and Verrucomicrobia decreased in the M group than the C group, while next-generation probiotic strain treatment markedly recovered the abundance of the above phyla (Figure 6i). At the genus level, we selected some representative taxa closely related to PD for analysis. We found that MPTP-induced PD mice greatly elevated the abundance of *Lactobacillus*, *Allobaculum*, and *Bifidobacterium* and reduced the abundance of *Oscillospira*, *Akkermansia*, *Sutterella*, *Coprococcus*, *Helicobacter*, *Desulfovibrio*, and *Ruminococcus*. Compared with the M group, the next-generation probiotic strain substantially restored the bacterial composition of PD mice at the genus level (Figure 6j). These results suggested that *L. lactis* MG1363-pMG36e-GLP-1 treatment would restore dysbacteriosis in PD mice.

## 4. Discussion

PD is the result of neurodegeneration and the death of DAergic neurons [19]; the number of PD patients could exceed 9.3 million by 2030 [2], which has become a serious public health problem. Nowadays, there is still no treatment to cure PD, which leads to an urgent demand to develop a neurotrophic therapy that can halt or reverse disease progression.

GLP-1 has shown neurotrophic effects in clinical trials in treating PD, yet the short half-life is still a noticeable limitation to the clinical application [13,14,15,31]. Previously, we had successfully constructed the genetically next-generation probiotics *L. lactis* MG1363-pMG36e-GLP-1 that possesses continuous expression of GLP-1 [18]. However, the possible influence of GLP-1 on ferroptosis in MPTP-induced mice models remained unknown. Herein, we used MPTP-induced PD mice models to explore the main role of *L. lactis* MG1363-pMG36e-GLP-1 as well as its potential therapeutic mechanism at the same time. MPTP is a protoxin, which can convert into a toxic MPP^+^ catalysed by MAO-B as it enters the brain, and is then transported into DAergic neurons to induce Parkinsonian changes [32]. Mice ingested MPTP showed severe behavioural dysfunctions, including reduced total movement distance, decreased exploratory ability, and increased latency time. We have found that *L. lactis* MG1363-pMG36e-GLP-1 can improve MPTP-induced behavioural dysfunction, which was similarly effective compared to liraglutide. Our results were accordant with the previous studies that *L. lactis* MG1363-pMG36e-GLP-1 could improve motor dysfunction in PD mice (Figure 1).

The major pathological changes of PD were the death of DAergic neurons and aggregated α-syn [6]. We found that *L. lactis* MG1363-pMG36e-GLP-1 abundantly promote the survival of TH^+^ neurons and inhibited aggregation of α-syn. Additionally, we found that the next-generation probiotic strain enhanced the activity of DAT, BDNF, and GDNF in PD mice. In summary, these results suggested that *L. lactis* MG1363-pMG36e-GLP-1 could thereby reverse the DAergic neurons’ death and synucleinopathy of PD model mice (Figure 2).

It was reported that the loss of integrity in the BBB and gut were important in the pathogenesis of PD [25]. Hence, we used H and E staining to evaluate the epithelial damage in the colon and SN. Collectively, our results have shown that *L. lactis* MG1363-pMG36e-GLP-1 treatment greatly attenuates histopathological changes in the SN and colon both. The intercellular space on the lateral surface is normally seamed by occluding junctions to guard the integrity of barriers [33]. Our findings suggested that the activity of the two dominant tight junction proteins in SN and colon is significantly down-expressed in the M group, including ZO-1, and occludin compared with the C group whereas next-generation probiotic strain treatment substantially restored such reductions. Our findings suggested that *L. lactis* MG1363-pMG36e-GLP-1 treatment potentially enhances the BBB and the intestinal barrier PD mice model (Figure 3 and Figure 6a–d).

Many studies suggest that ferroptosis participated in the pathophysiology of PD, which is manifested by mitochondrial damage [7]. The ultrastructural changes of PD mice showed that the mitochondria were diminished numerically and dwindled morphologically, which accorded to manifestations of ferroptosis [34]. While *L. lactis* MG1363-pMG36e-GLP-1 could recover the mitochondrial morphology. Meanwhile, we further proved that *L. lactis* MG1363-pMG36e-GLP-1 can inhibit MPTP-induced iron deposition. DMT1 and TfR1 are two major iron transporters from the blood into peripheral tissue cells [35]. Fe^3+^ binds to TfR1, when the pH changes, it will release as well as convert the Fe^3+^ to Fe^2+^, and then be transported into the cytoplasm via DMT1 [36]. We found that up-regulation of DMT1 in PD mice could be suppressed by *L. lactis* MG1363-pMG36e-GLP-1, indicating that the iron-suppression of *L. lactis* MG1363-pMG36e-GLP-1 might be mediated via suppression of DMT1, instead of TfR1, which is consistent with the previous report [34]. Our findings revealed that *L. lactis* MG1363-pMG36e-GLP-1 further inhibited ferroptosis of PD mice models by modulating the Keap1-Nrf2-GPX4 pathway. Among them, GPX4 is a necessary protein that suppresses lipid hydroperoxides on membranes, suggesting its dominating role in the ferroptotic signalling pathway [27]. In addition, the expression levels of Keap1-Nrf2 are considered to represent a preventive and therapeutic agent for a variety of diseases substantially related to oxidative stress [37,38,39]. Herein, we found that *L. lactis* MG1363-pMG36e-GLP-1 can inhibit ferroptosis via potentiating the Keap1-Nrf2-GPX4 signalling pathway. Furthermore, it is reported that the redox states homeostasis was controlled by the FSP1-NADH-CoQ10 axis, which acts as an independent pathway with a GPX4 pathway to inhibit ferroptosis [40,41]. FSP1 preserves cells from ferroptosis via inhibiting CoQ10, a strong antioxidant to suppress lipid peroxidation. We found *L. lactis* MG1363-pMG36e-GLP-1 is able to inhibit ferroptosis by promoting FSP1 expression, thereby reducing lipid peroxidation and exerting neuroprotective effects in PD mice. Additionally, we also evaluated the activity of ACSL4, which seemed to have a significant role in ferroptosis in the nascent study [42]. The data in our study have demonstrated that *L. lactis* MG1363-pMG36e-GLP-1 can inhibit ferroptosis via down-regulating ACSL4. Collectively, these results supported that ferroptosis take part in the death of DAergic neurons (Figure 4).

Oxidative stress seemed to play an important role in DAergic nerve cell death in PD, which is related to ferroptosis [43]. We identified that MPTP increases the levels of ROS, whereas *L. lactis* MG1363-pMG36e-GLP-1 can effectively decrease the level of ROS. Oxidative stress contributes greatly to PD and is extensively found in patients and PD models [44,45]. MDA is the main terminal product of lipid peroxidation, and its changes could reflect tissue peroxidation damage [46]. We observed that MPTP treatment augmented the level of MDA, whereas *L. lactis* MG1363-pMG36e-GLP-1 decreased the activity of MDA. Antioxidants can neutralise free radicals before triggering chain reactions that could attack vital molecules for physiological processes. The major function of SOD is to catalyze superoxide radicals (O_2_^−^) and protect tissue from its deleterious effects. Additionally, GSH-Px is another oxidant protector by catalyzing the reduction of H_2_O_2_ [47]. The GSH-Px and SOD levels of PD mice were greatly reduced. However, after *L. lactis* MG1363-pMG36e-GLP-1 treatment the GSH-Px and SOD activities in the brain were salvaged, suggesting that *L. lactis* MG1363-pMG36e-GLP-1 shows strong antioxidant capacity. In conclusion, compared with liraglutide and wild-type *L. lactis* MG1363, *L. lactis* MG1363-pMG36e-GLP-1 could more effectively reduce oxidative stress in PD model mice (Figure 5).

Through microbiota-gut-brain axis, gut microbiome could affect not just gastrointestinal physiology that is in the vicinity, but the CNS (central nervous system) function as well [48]. To assess the effect of next-generation probiotic strain on intestinal microecology, we performed high-throughput sequencing. We found that *L. lactis* MG1363-pMG36e-GLP-1 slightly recovered α-diversity and β-diversity of intestinal microbiota in PD mice. The Venn plot also showed that *L. lactis* MG1363-pMG36e-GLP-1 restored the bacterial diversity to a certain extent. Herein, the abundance of several bacteria at genus level increased, including *Lactobacillus*, *Allobaculum*, and *Bifidobacterium*, and that of *Akkermansia*, and *Coprococcus* were decreased during the progression of PD. Furthermore, such dysbiosis could be restored by treating with *L. lactis* MG1363-pMG36e-GLP-1. Unfortunately, based on current research, the correlation between *Lactobacillus* and PD is still unknown, and this requires future studies. However, it is reported that the increase of *Lactobacillus* in PD patients might be attributed to frequent constipation [49]. Notably, the abundance of *Akkermansia* was decreased in the M group and elevated after *L. lactis* MG1363-pMG36e-GLP-1 treatment. The role of *Akkermansia* is not yet clear but maintaining constancy of *Akkermansia* may be a prerequisite for gut physiological functioning [50]. Some studies reported that *Akkermansia muciniphila* is a potential probiotic that exerts beneficial effects on the intestinal mucosal layer [51]. Hence, low *Akkermansia* abundance could potentially impair intestinal mucosal integrity and allow virulence factors such as lipopolysaccharide (LPS) to penetrate the epithelial barrier. Further investigations are required (Figure 6e–j).

## 5. Conclusions

Our results showed that next-generation probiotic strain *L. lactis* MG1363-pMG36e-GLP-1 has great neurotrophic potential in treating MPTP-induced PD mice models, which is attributed to the suppression of the ferroptotic signalling pathway. Additionally, our findings suggest that the reduced oxidative stress as well as lipid peroxidation, decreased iron deposition, and restoration of intestinal microbial diversity via *L. lactis* MG1363-pMG36e-GLP-1 are of significance for inhibiting ferroptosis, providing a putative pharmaceutic drug for clinical prophylaxis and treating PD. Nevertheless, the limited number of experimental animals in the current study is a non-negligible influence for us to deduce a better statistical conclusion.

## Data Availability

The raw data of 16S rRNA high-throughput sequencing was uploaded to the Sequence Read Archive (SRA) database of NCBI (PRJNA880461). Other data will be made available by the authors, without undue reservation.

## Supplementary Materials

The following supporting information can be downloaded at: https://www.mdpi.com/article/10.3390/nu14224886/s1, Table S1: Antibodies used in this study.
