# Supplementary material for: Neurotrophic Role of the Next-Generation Probiotic Strain L. lactis MG1363-pMG36e-GLP-1 on Parkinson’s Disease via Inhibiting Ferroptosis
Source: Nutrients. 2022 Nov 18;14(22):4886. doi: 10.3390/nu14224886 (PMC9698534; doi:10.3390/nu14224886)
Supplement: Supplementary file 1 [file nutrients-14-04886-s001.zip › nutrients-1999018-supplementary.pdf]

Table S1. Antibodies used in this study.

Antibodies	Source/Cat. No.	Host	Dilution
Tyrosine hydroxylase (TH)	Proteintech (25859-1-AP)	Rabbit	1 : 5, 000 (WB)<br>1 : 1, 000 (IHC)
α-synuclein (α-syn)	Proteintech (10842-1-AP)	Rabbit	1 : 1, 000 (WB)<br>1 : 200 (IF)
Brain-derived neurotrophic factor (BDNF)	Servicebio (GB11559)	Rabbit	1 : 100 (IF)
Glial cell line-derived neurotrophic factor (GDNF)	Servicebio (GB11403)	Rabbit	1 : 200 (IF)
Glutathione peroxidase 4 (GPX4)	Abcam (ab125066)	Rabbit	1 : 5, 000 (WB)<br>1 : 200 (IF)
Zonula occludens-1 (ZO-1)	Proteintech (21773-1-AP)	Rabbit	1 : 500 (WB)
Occludin	Proteintech (13409-1-AP)	Rabbit	1 : 1, 000 (WB)
β-actin	CST (4970S)	Mouse	1 : 1, 000 (WB)
DAT	Proteintech (22524-1-AP)	Rabbit	1 : 1, 000 (WB)
GLP-1 receptor (GLP-1R)	Proteintech (26196-1-AP)	Rabbit	1 : 1, 000 (WB)
Transferrin receptor 1 (TfR1)	Abcam (ab84036)	Rabbit	1 : 1, 000 (WB)
Divalent metal transporter 1 (DMT1)	Abcam (ab55735)	Mouse	1 : 1, 000 (WB)
Ferroptosis suppressor protein 1 (FSP1)	Millipore (07-2274)	Rabbit	1 : 1, 000 (WB)
Long-chain acyl-CoA synthetase 4 (ACSL4)	Santa Cruz Biotechnology (sc-365230)	Mouse	1 : 1, 000 (WB)
Kelch-like ECH-associated protein 1 (Keap1)	Proteintech (60027-1-lg)	Mouse	1 : 2, 000 (WB)
Nuclear factor-like 2 (Nrf2)	CST (12721S)	Rabbit	1 : 1, 000 (WB)
